# Clinical Learning-Curve Evidence and Experience-Related Temporal Trends of Resuscitative Endovascular Balloon Occlusion of the Aorta in Patients with Trauma: A Systematic Review

**DOI:** 10.3390/jcm15186959

**Published:** 2026-09-08

**Authors:** Bayarjargal Gavaadorj, Soon Ki Min, Youngmin Kim, Kang Kook Choi, Byungchul Yu, Gil Jae Lee, Dong Keon Yon, Wu Seong Kang

**Affiliations:** 1 Department of Trauma Surgery, Gachon University Gil Medical Center, Incheon 21565, Republic of Korea; bayarjargalgavaadorj@gmail.com (B.G.); minsk0611@gilhospital.com (S.K.M.); skyofmay@gilhospital.com (Y.K.); calvin511@naver.com (K.K.C.); kane2123@gilhospital.com (B.Y.); nonajugi@gilhospital.com (G.J.L.); 2 Department of Traumatology, Gachon University College of Medicine, Incheon 21565, Republic of Korea; 3 Center for Digital Health, Medical Science Research Institute, Kyung Hee University Medical Center, Kyung Hee University College of Medicine, Seoul 02447, Republic of Korea; 4 Department of Pediatrics, Kyung Hee University College of Medicine, Seoul 02447, Republic of Korea

**Keywords:** wounds and injuries, REBOA, systematic review, learning curve

## Abstract

**Background/Objectives**: Resuscitative endovascular balloon occlusion of the aorta (REBOA) is a technically demanding, low-frequency intervention that requires both operator proficiency and coordinated trauma-system performance. We systematically reviewed direct clinical evidence on REBOA learning curves and indirect experience-related temporal trends. **Methods**: PubMed/MEDLINE, Embase, and the Cochrane Library were searched through 24 April 2026. The prespecified primary outcomes were procedural efficiency and safety; mortality and survival were secondary outcomes. The review protocol was prospectively registered in PROSPERO (CRD420261357432). Because of substantial clinical and methodological heterogeneity, quantitative pooling was not performed, and the evidence was synthesized narratively. Studies were classified as direct case-based learning-curve analyses using sequential case numbers or cumulative procedural volume, or as indirect temporal-trend analyses using calendar year or successive study periods as proxies for institutional or system-level experience. **Results**: Seven observational studies were included. Three studies provided direct case-based learning-curve evidence using sequential case numbers, whereas four registry-based studies evaluated calendar-time trends as indirect proxies for institutional or system-level experience. The three direct studies identified study-specific inflection points at different case numbers, including approximately 5 and 10 cases for individual- and institutional-level technical challenges and 5, 17, and 34 cases for risk-adjusted mortality across three of five centers; improvement was not consistently observed across all centers. In contrast, the four temporal-trend studies generally reported improved outcomes over time but did not identify case-volume thresholds and could not isolate the effect of accumulated experience from concurrent changes in trauma care. **Conclusions**: Direct clinical evidence on the REBOA learning curve remains limited to a small number of case-based studies. Calendar-time trends may reflect broader institutional or system-level maturation but cannot independently establish a learning-curve effect. By providing an integrated clinical perspective on how REBOA proficiency may develop in real-world practice, this review highlights the need for prospective studies using standardized procedural outcomes and risk-adjusted learning-curve methods.

## 1. Introduction

Non-compressible torso hemorrhage (NCTH) remains a leading cause of potentially preventable death after severe trauma [1]. Because bleeding within the torso cannot generally be controlled by direct external compression, survival depends on rapid resuscitation and timely definitive hemorrhage control [2,3]. Resuscitative endovascular balloon occlusion of the aorta (REBOA) provides temporary aortic occlusion to reduce distal hemorrhage and support perfusion proximal to the balloon. In carefully selected patients with life-threatening subdiaphragmatic hemorrhage, REBOA may therefore serve as a temporizing adjunct or bridge to definitive operative or endovascular hemorrhage control [4,5,6].

Despite its physiological rationale, the clinical benefit of REBOA remains uncertain. Contemporary randomized and synthesized evidence has not established a consistent survival advantage and has raised concerns regarding potential harm [7,8]. This heterogeneity may reflect differences in patient selection, device and occlusion strategies, timely access to definitive hemorrhage control, and institutional readiness—including operator and team experience—rather than the effect of aortic occlusion alone [7,8,9,10].

Experience is therefore a plausible modifier of REBOA performance and outcomes. REBOA proficiency is both operator- and system-dependent: technical success requires reliable vascular access and balloon control, while effective implementation also depends on coordinated progression from deployment to definitive hemostasis [9,10]. Importantly, REBOA is primarily a bridging intervention rather than definitive hemorrhage control. Its clinical effectiveness therefore depends not only on appropriate patient selection and successful balloon deployment but also on the ability of the trauma system to rapidly transition the patient to definitive hemostatic procedures, such as surgery or angioembolization. Consequently, maturation of the multidisciplinary trauma system—including coordinated decision-making, procedural readiness, and timely access to definitive hemorrhage control—may be as important as technical proficiency of the individual operator. Learning should therefore be considered at both the individual and institutional levels. Technical challenges have been observed predominantly during the early case experience of both individual operators and institutions, and higher-utilization centers have reported shorter times to REBOA placement [11,12]. Because REBOA is a low-frequency, high-acuity intervention and competence may decline without continued exposure, its learning curve should be considered across individual, institutional, and broader trauma-system levels [13]. However, these concepts are related but not equivalent. Individual operator learning reflects changes in procedural performance as a specific operator accumulates cases, whereas institutional learning reflects the collective experience and coordination of the multidisciplinary team and may be related to, but is not synonymous with, institutional procedural volume. Improvement over calendar time is conceptually distinct because it may reflect broader system maturation as well as concurrent changes in patient selection, devices, procedural strategies, training, and trauma-system organization. Therefore, temporal improvement alone cannot establish a procedural learning-curve effect. Previous reviews have addressed clinical effectiveness, complications, or simulation-based training, but no systematic review has integrated clinical learning-curve evidence across these levels with procedural and patient outcomes or examined whether reported proficiency estimates are generalizable [8,9,14].

Accordingly, this systematic review aimed to investigate two complementary forms of evidence: direct case-based analyses of operator- or institution-level learning curves and indirect calendar-time trends potentially reflecting broader institutional or trauma-system maturation. We evaluated how accumulated experience was defined and analyzed, whether increasing experience was associated with procedural efficiency and safety, and whether direct case-based studies supported a reproducible case-volume threshold. Calendar-time trends in mortality, survival, and other clinical outcomes were additionally examined as indirect indicators of evolving institutional or system-level performance.

## 2. Materials and Methods

### 2.1. Literature Search

This systematic review was conducted in accordance with the Preferred Reporting Items for Systematic Reviews and Meta-Analyses (PRISMA) 2020 guidelines [15] (Supplementary Material). The study protocol was prospectively registered in the International Prospective Register of Systematic Reviews (PROSPERO; CRD420261357432). The methodological quality of the present study was assessed using the A MeaSurement Tool to Assess systematic Reviews (AMSTAR 2) tool (Supplementary Material) [16]. A systematic literature search was performed in MEDLINE (via PubMed), Embase, and the Cochrane Library to identify eligible studies published through 24 April 2026. The search strategy was developed a priori using a combination of controlled vocabulary (Medical Subject Headings [MeSH] and Emtree terms) and free-text keywords (Supplementary Table S1). To ensure comprehensive study identification, the reference lists of eligible articles and relevant review papers were also manually searched.

### 2.2. Eligibility Criteria

Studies were eligible if they met the following criteria: (1) included adult patients (≥18 years) undergoing REBOA for traumatic hemorrhage; (2) evaluated accumulated REBOA experience using either direct measures, such as sequential case number or cumulative procedural volume, or indirect temporal proxies, such as calendar year or successive study periods, at the operator, institutional, multicenter, or national level: temporal-trend studies were eligible only when they explicitly compared REBOA practices, procedural outcomes, or clinical outcomes across ordered time periods within the same institution, registry, or healthcare system; (3) compared early with later experience within the same cohort, although single-arm learning-curve analyses were also eligible; (4) reported at least one relevant learning-curve outcome, including procedural efficiency, technical success, procedure-related complications, clinical outcomes, or a case-number threshold or inflection point for proficiency; and (5) were original clinical studies reporting either a direct case-based learning-curve analysis or an experience-related temporal-trend analysis were eligible. Studies involving pediatric patients (<18 years), non-traumatic indications for REBOA, case reports or case series including fewer than five patients, conference abstracts, review articles, systematic reviews or meta-analyses, non-clinical studies, studies without a learning-curve or cumulative-experience analysis, and non-English-language publications were excluded.

### 2.3. Definition and Classification of Accumulated Experience

Accumulated experience was classified into two categories. Direct learning-curve evidence was defined as analyses using sequential case number, cumulative procedural volume, or another case-based measure of experience. Indirect temporal evidence was defined as analyses using calendar year or successive study periods as proxies for institutional or system-level experience. Because calendar time may also capture changes in patient selection, devices, resuscitation strategies, and trauma-system organization, temporal-trend studies were not considered direct evidence of procedural learning and were not used to derive proficiency thresholds.

### 2.4. Study Selection

Records identified through the database search were imported into EndNote version 2025 (Clarivate, Philadelphia, PA, USA), where duplicate records were removed automatically and any remaining duplicates were removed manually. Two reviewers (GB and SKM) independently screened titles and abstracts, followed by full-text assessment of potentially eligible studies. Disagreements were resolved by consensus or, when necessary, by consultation with a third reviewer (YK).

### 2.5. Outcomes

The prespecified primary outcomes were procedural efficiency and procedural safety. Procedural efficiency included procedure-related time metrics and technical success, whereas procedural safety included REBOA-related complications, such as access-site vascular injury, arterial dissection, pseudoaneurysm, limb ischemia, and the need for vascular repair. Secondary outcomes included mortality, survival, transfusion requirements, physiological changes, and other clinical outcomes evaluated in relation to accumulated REBOA experience. Case-volume thresholds, inflection points, and other estimates indicating operator- or institution-level proficiency were additionally extracted as key learning-curve measures.

### 2.6. Data Extraction

Data were extracted independently by two investigators (GB and SKM) using a standardized, pilot-tested data extraction form. Extracted data included study characteristics (first author, publication year, country, study design, study period, sample size, and study population); learning-curve characteristics (level of experience assessed, analytical method, definition of accumulated experience, and proficiency threshold); prespecified procedural outcomes, including procedural time, technical success, and procedure-related complications; and secondary clinical outcomes, including mortality and survival. Case-volume thresholds and inflection points indicating proficiency were also extracted. Evidence type was categorized as direct case-based learning-curve evidence or indirect temporal evidence. The measure of accumulated experience was extracted separately as sequential case number, cumulative procedural volume, calendar year, or successive study period. Disagreements were resolved by consensus or, when necessary, through consultation with a third investigator (YK).

### 2.7. Quality Assessment

The methodological quality of the included studies was independently assessed by two investigators (GB and SKM) using the Newcastle–Ottawa Scale (NOS) [17]. Because the included studies comprised heterogeneous observational designs that did not uniformly correspond to conventional comparative non-randomized studies of interventions, the NOS was used as a structured framework for methodological appraisal. Items that were not applicable to a particular study design were designated as not available (N/A) rather than considered satisfied. In particular, the selection item for the non-exposed cohort (S2) was considered N/A when no independently selected comparator cohort was present. Given these design differences, aggregate NOS scores were not calculated, and methodological limitations were interpreted at the item and domain levels. Any disagreements were resolved through discussion and consensus or, when necessary, by consultation with a third reviewer (YK).

### 2.8. Data Synthesis

Findings were first stratified according to the directness of the experience measure. Studies using sequential case numbers or cumulative procedural volume were synthesized as direct learning-curve evidence, whereas studies using calendar time or successive study periods were synthesized separately as indirect experience-related temporal evidence. Quantitative synthesis was not performed because the included studies differed substantially in their definitions of accumulated experience, levels of learning-curve assessment, outcome definitions, and analytical methods. In particular, the prespecified primary procedural outcomes were inconsistently reported and could not be harmonized across studies. Therefore, all findings were synthesized narratively according to the level of assessment, analytical approach, and reported outcome domain.

### 2.9. Deviations from the Registered Protocol

Several deviations from the registered protocol were made because of the characteristics of the eligible evidence. First, the Newcastle–Ottawa Scale was used instead of the initially planned RoB 2 and ROBINS-I because the included evidence consisted of observational learning-curve, registry-based, and temporal-trend studies that did not uniformly evaluate a clearly defined intervention effect against a concurrent comparator. Second, the planned meta-analysis and meta-regression were not performed because of substantial clinical and methodological heterogeneity in the definitions of accumulated experience, outcome measures, and analytical approaches. The prespecified outcome hierarchy was otherwise maintained, with procedural efficiency and safety considered primary outcomes and mortality and survival considered secondary outcomes. Third, the registered protocol did not explicitly distinguish direct case-based measures of accumulated experience from calendar-time proxies. During full-text assessment, we made a post hoc decision to include studies using calendar year or successive-period analyses as a separate category of indirect evidence of institutional or system-level experience. This decision was made before final study inclusion, classification, and data synthesis. These studies were subsequently synthesized separately from direct case-based learning-curve studies.

## 3. Result

### 3.1. Study Selection

**Figure 1:**
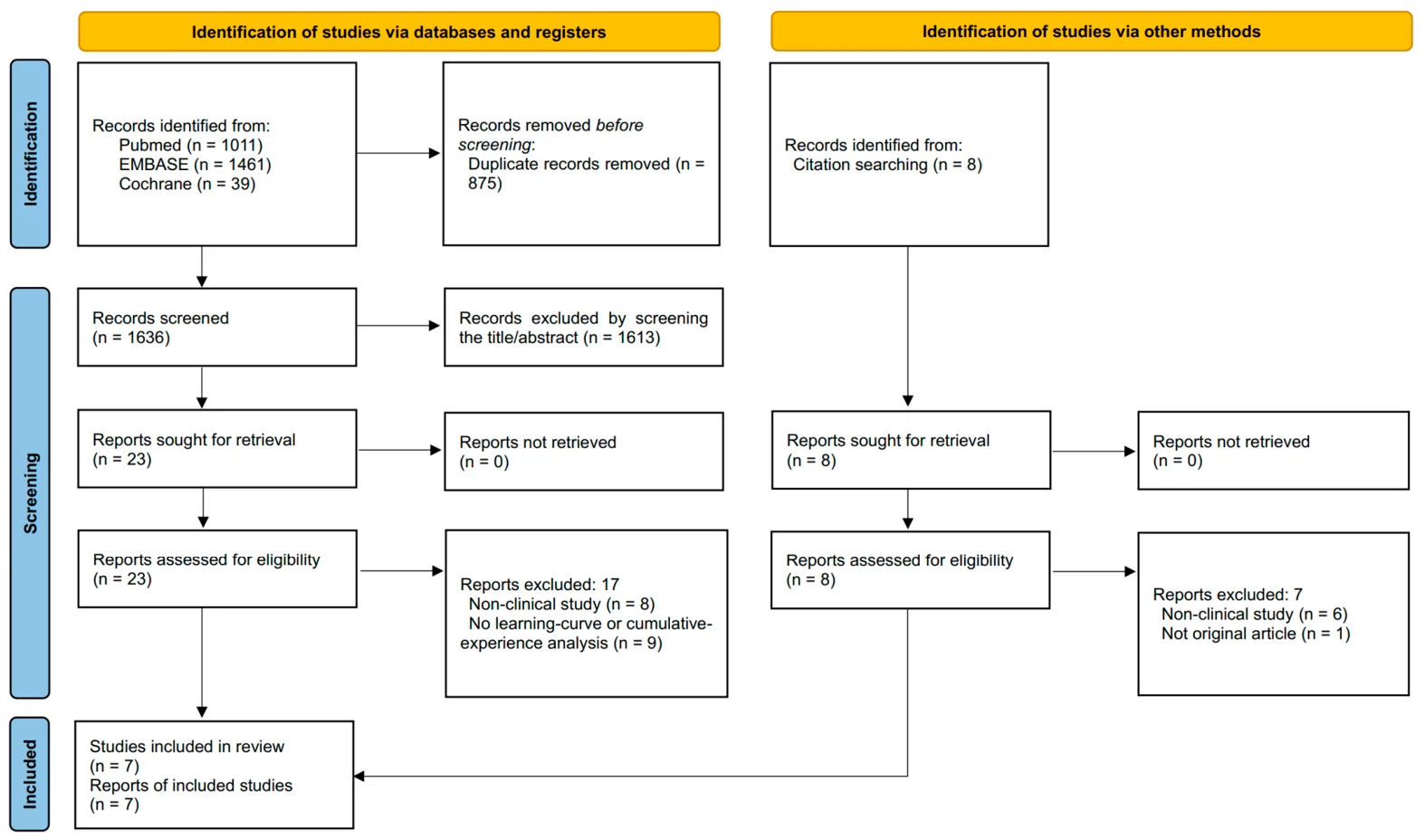
Flowchart of study selection.

EndNote identified 875 duplicate records. Following duplicate removal, a total of 1636 records remained for title and abstract screening. During this stage, 1613 records were excluded for the following reasons: non-original articles (k = 536), not human studies (k = 227), ex vivo studies (k = 1), insufficient information to determine eligibility (k = 211), non-English-language publications (k = 28), and ineligible populations or interventions (k = 544). During title and abstract screening, an additional 66 residual duplicate records were identified and excluded. An additional eight studies were identified through hand-searching. The full texts of the remaining 31 articles were then evaluated in detail, resulting in the exclusion of 24 studies [7,12,13,14,18,19,20,21,22,23,24,25,26,27,28,29,30,31,32,33,34,35,36,37]. Reasons for exclusion at this stage included non-clinical studies (k = 14), systematic reviews (k = 1), and no learning-curve or cumulative-experience analysis (k = 9). Ultimately, seven studies [11,38,39,40,41,42,43] met the operationalized eligibility criteria and were included in the final qualitative synthesis (Figure 1).

### 3.2. Included Study Characteristics

A total of seven studies [11,38,39,40,41,42,43] were included in this systematic review (Table 1). The studies were published between 2020 and 2026 and were conducted in Japan (k = 2), the United States (k = 3), and the Republic of Korea (k = 2). All included studies were observational. Sample sizes ranged from 31 to 2557 patients. Study populations comprised trauma patients undergoing REBOA at institutional, multicenter, or nationwide levels. Three studies [11,40,43] directly evaluated cumulative case experience, whereas four [38,39,41,42] assessed experience-related temporal trends using calendar year or successive study periods. Direct learning-curve methods included cumulative sum (CUSUM), risk-adjusted CUSUM, and cubic regression. Indirect temporal analyses primarily used regression-based comparisons across calendar periods. The outcomes reported across the included studies included procedural performance, in-hospital mortality, survival, procedure-related complications, and case-volume thresholds for proficiency.

### 3.3. Level and Approach of Learning-Curve Assessment

The REBOA learning curve was evaluated across three analytical levels. Four studies assessed learning at the national or multicenter registry level [38,39,41,42], two at the institutional level [40,43], and one at both the individual surgeon and institutional levels [11]. Two analytical approaches were identified. Four studies used procedure year as a surrogate for accumulated experience and evaluated outcomes using temporal-trend regression without applying a formal learning-curve model [38,39,41,42]. In contrast, three studies used sequential case numbers to model cumulative experience, applying either cumulative sum (CUSUM) analysis [40,43] or cubic regression [11].

### 3.4. Procedural Efficiency and Safety Outcomes

The prespecified primary outcomes of procedural efficiency and safety were reported inconsistently across the included studies. Procedural performance was assessed primarily using sequential case-number analyses. Choi et al. [40] evaluated aortic occlusion time, whereas Moore et al. [11] examined operator-reported technical challenges. Kim et al. [43] used cumulative sum (CUSUM) methods to assess procedural performance across participating centers. The evaluated outcomes included risk-adjusted mortality, aortic occlusion time, time from common femoral artery access to REBOA placement, door-to-balloon time, and time from injury to hospital admission. Regarding procedural complications and technical challenges, outcomes varied across studies. In a five-year registry analysis, Bukur et al. [39] reported that the overall REBOA-specific complication rate remained stable at 4.5% (*p* = 0.575). The rate of extremity ischemia varied over time, ranging from 0% to 2.9% (*p* = 0.047), whereas the need for surgical patch angioplasty decreased from 2% to 0% (*p* = 0.046). Moore et al. [11] evaluated technical challenges rather than clinical complications and found that catheter removal was the most frequently reported technical challenge, accounting for 37.0% of individual-level and 46.2% of institutional-level challenges. Choi et al. [40] reported that one patient (3.2%) required hemodialysis for acute kidney injury, while no limb ischemia events were observed (0%). In contrast, the remaining studies did not report temporal or experience-based trends in REBOA-related complications [38,41,42,43]. Overall, procedural safety outcomes were reported inconsistently and were generally not evaluated in relation to cumulative procedural experience, precluding meaningful synthesis of a safety-related learning curve.

### 3.5. Secondary Clinical Outcomes

Mortality or survival, which were prespecified as secondary outcomes in this review, were evaluated in six [38,39,40,41,42,43] of the seven included studies and were the most frequently reported clinical outcomes. Four registry-based studies [38,39,41,42] assessed temporal changes in in-hospital mortality or survival, whereas two case-based studies [40,43] incorporated mortality into formal learning-curve analyses.

### 3.6. Direct Case-Based Learning-Curve Evidence

Three studies [11,40,43] provided direct case-based evidence by modeling performance against sequential REBOA case numbers. All three [11,40,43] identified a case-number range associated with improved performance, although the thresholds varied according to the outcome, analytical method, and level of assessment. Choi et al. [40] reported an inflection point at the 17th consecutive case based on non-risk-adjusted CUSUM analysis of mortality and aortic occlusion time. Kim et al. [43] used RA-CUSUM across five centers and observed improving performance after 5 to 34 cases in three centers, whereas the remaining centers showed no consistent improvement. Moore et al. [11] found that technical challenges were concentrated in the early learning phase, with proficiency achieved after approximately five cases for individual surgeons and ten cases at the institutional level. These case-number estimates were derived from different outcomes and analytical methods and therefore should not be interpreted as equivalent or validated thresholds for overall REBOA proficiency.

### 3.7. Temporal Trends at the Registry and System Level

All four registry-based studies [38,39,41,42] reported improved clinical outcomes over calendar time. These findings were considered indirect evidence of evolving institutional or system-level performance rather than direct evidence of a procedural learning curve. Aoki et al. [38] reported a stepwise increase in in-hospital survival from 39% in 2004–2007 to 49% in 2008–2011 and 60% in 2012–2015. Compared with the early period, adjusted survival was significantly higher in the late period (OR, 2.98; 95% CI, 1.62–5.48), but not in the mid period (OR, 1.61; 95% CI, 0.90–2.90). Bukur et al. [39] found a 22% annual reduction in adjusted mortality, accompanied by evolving procedural practices and temporal changes in selected vascular complications, including a decrease in the need for surgical patch angioplasty from 2% to 0% (*p* = 0.046). Similarly, Hoshi et al. [41] reported that in-hospital mortality decreased from 91.3% to 50.9% between 2004 and 2021, with an overall mortality of 59.7%, while adjusted mortality odds also declined over time. Rayzah et al. [42] reported a decrease in unadjusted mortality from 57.8% (48/83) in 2019 to 36.5% (35/96) in 2022, although this temporal trend may have been influenced by concurrent changes in patient characteristics and trauma-system practices. None of these studies evaluated outcomes in relation to cumulative case numbers or identified a proficiency threshold.

### 3.8. Impact of Risk Adjustment

Risk adjustment relevant to the association between accumulated experience and outcomes was performed in four studies [38,39,41,43]. Rayzah et al. [42] performed multivariable mortality-predictor analysis, but its temporal mortality trend was not risk-adjusted. Choi et al. [40] and Moore et al. [11] did not perform risk adjustment. Among the temporal trend studies, improvements persisted after adjustment for injury severity and admission physiology in several analyses. However, such adjustment cannot fully separate accumulated REBOA experience from concurrent changes in patient selection, device technology, occlusion strategy, definitive hemorrhage control, or trauma-system organization. Kim et al. [43] were the only investigators to incorporate patient-level risk adjustment directly into a formal CUSUM learning-curve model. Improvement was observed in only three of five centers, highlighting substantial between-center heterogeneity and suggesting that accumulated case experience alone may not fully explain institutional performance. Risk-adjusted CUSUM analysis was not feasible in the study by Choi et al. [40] because of the small sample size.

### 3.9. Excluded Studies

Twenty-four reports were excluded after full-text review, primarily for the reasons summarized in Supplementary Table S2. Most (*n* = 14) were simulation, cadaveric, or animal studies that evaluated skill acquisition or procedural performance rather than clinical outcomes and therefore did not assess patient-based learning curves. One systematic review was excluded because of its ineligible publication type; however, its reference list was hand-searched to identify additional eligible studies.

### 3.10. Methodological Quality Assessment

Methodological appraisal demonstrated substantial variation across individual NOS items and domains (Table 2). Because none of the included studies had an independently selected non-exposed cohort, S2 was considered not applicable for all studies. The principal methodological concerns involved comparability and control for confounding, particularly in non-risk-adjusted learning-curve analyses and calendar-time studies susceptible to concurrent changes in patient selection and clinical practice. Given the heterogeneity of study designs, aggregate NOS scores were not calculated or interpreted as measures of overall study quality.

## 4. Discussion

### 4.1. Key Findings

Only three [11,40,43] of the seven included studies directly evaluated the REBOA learning curve using sequential case numbers. The three direct studies reported heterogeneous, study-specific inflection points at different case numbers, based on different outcomes and analytical approaches. The remaining four studies [38,39,41,42] evaluated calendar-time trends at registry or system levels. Although these studies generally reported improved survival or mortality over time, calendar year is an indirect proxy that cannot distinguish accumulated REBOA experience from concurrent advances in patient selection, devices, resuscitative practice, and trauma-system organization. Taken together, these findings provide an initial clinical perspective on how REBOA proficiency may develop with accumulated experience in real-world trauma practice. However, these findings should be interpreted with substantial caution. Direct case-based evidence was limited to three observational studies, and the reported case-number inflection points were derived from heterogeneous outcomes, analytical methods, and levels of assessment, including operator-reported technical challenges, procedural time-related measures, and mortality-based outcomes. Patient-level risk adjustment was limited, and improvement was not consistently observed across centers or across procedural and clinical outcomes. Consequently, the reported case numbers are not directly comparable and should not be interpreted as defining a generalizable learning range. Moreover, none of these estimates has been externally validated. The indirect calendar-time evidence is subject to additional uncertainty because calendar time is an imperfect surrogate for accumulated experience and temporal trends may reflect concurrent changes in patient selection, devices, procedural strategies, and trauma-system organization. Accordingly, confidence in the available evidence is very limited for both direct case-based learning-curve evidence and indirect calendar-time evidence. The case-number estimates should therefore be considered study-specific, outcome-dependent, and hypothesis-generating rather than validated proficiency, competency, or credentialing thresholds. The current evidence is insufficient to support a specific case-number requirement for clinical implementation.

### 4.2. Comparison with Previous Research

The learning-curve findings of this review provide important context for interpreting recent clinical studies reporting unfavorable or inconsistent outcomes with REBOA. The UK-REBOA trial [7], the only randomized trial of REBOA in trauma, provides an important contrast to observational learning-curve studies. Balloon inflation was achieved in only 19 of 46 patients assigned to REBOA, and definitive hemorrhage control was delayed compared with standard care. Because the trial used an intention-to-treat design, its mortality estimate reflects the overall REBOA strategy, including failed access, procedural interruption, and treatment delays, rather than successful aortic occlusion alone. A post hoc sensitivity analysis excluding the first participant randomized to the REBOA strategy at each site did not materially alter the overall findings [7]; however, this analysis did not directly assess individual operator experience or cumulative procedural volume. Therefore, the extent to which the learning curve influenced the trial outcomes remains uncertain, and the unfavorable findings may have reflected multiple interacting factors, including patient selection, protocol adherence, ischemic burden, procedural experience, and system-level delays.

Engberg et al. reviewed REBOA training primarily in simulation settings using virtual-reality simulators, task trainers, porcine models, and cadavers [14]. Training generally improved procedural time and assessed technical performance, but most studies were uncontrolled, used nonvalidated assessment tools, and did not examine skill transfer or patient outcomes. In contrast, the present review included only patient-based clinical studies evaluating accumulated experience in relation to procedural performance, complications, and mortality. These reviews are therefore complementary: simulation evidence supports technical skill acquisition, whereas clinical evidence suggests that proficiency may additionally depend on patient selection, team coordination, and timely definitive hemorrhage control.

Recent evidence has not demonstrated a consistent clinical benefit of REBOA. Harfouche et al. [8], in their meta-analysis, found no significant mortality advantage over no REBOA in hemodynamically unstable trauma patients and reported higher mortality among patients with pelvic fractures. However, confidence in the available evidence is very limited because of selection bias, substantial heterogeneity, and imprecision. These findings may reflect not only the limitations of REBOA itself but also variability in patient selection, procedural timing, operator experience, and institutional coordination. Differences in device profiles, occlusion strategies, outcome definitions, and trauma-system organization may further contribute to the heterogeneous findings across clinical and registry-based studies. Taken together with the randomized and training literature discussed above, the findings of the present review suggest that improvements observed with increasing experience or calendar time cannot be attributed solely to operator-level procedural learning. Because REBOA serves primarily as a bridge to definitive hemorrhage control, clinical outcomes may also depend on the efficiency of subsequent surgical or endovascular hemostasis and broader multidisciplinary system maturation. Thus, the observed trends may reflect a combination of procedural learning and institutional or system-level maturation, although their relative contributions cannot be determined from the available evidence. This hypothesis requires prospective evaluation using direct measures of both operator and institutional experience.

### 4.3. Clinical Implications of Present Study

The available evidence suggests that REBOA proficiency may depend not only on the technical skills of individual operators but also on the preparedness and coordination of the broader trauma team. Because REBOA is performed infrequently in many institutions, opportunities to acquire and maintain proficiency through clinical exposure alone may be limited. Simulation-based training, standardized procedural workflows, and regular multidisciplinary review may therefore be reasonable components of institutional REBOA programs. In addition, performance-monitoring approaches such as CUSUM analysis may help identify changes in procedural performance over time and support targeted retraining when necessary. However, the available evidence remains insufficient to determine specific credentialing requirements, standardized training models, or minimum procedural-volume thresholds. The reported case-number estimates should therefore be interpreted as preliminary indicators rather than definitive standards for practice. Calendar-time improvements should not be interpreted as evidence that a specific number of cases ensures competence. Rather, they suggest that REBOA outcomes may evolve alongside broader institutional maturation, including protocol development, multidisciplinary coordination, device adoption, and improved access to definitive hemorrhage control.

### 4.4. Future Research Directions

Future studies should evaluate REBOA learning curves prospectively using standardized and clinically relevant outcome definitions. Procedural efficiency should be assessed with clearly defined time intervals, such as access-to-balloon inflation time and time from emergency department arrival to definitive hemorrhage control, while procedural safety should include technical failure, access-site complications, limb ischemia, and the need for vascular intervention. Risk-adjusted analyses are also needed to distinguish the association of accumulated experience from differences in injury severity, physiological status, patient selection, and institutional practice. Multilevel risk-adjusted CUSUM or hierarchical models may help distinguish operator learning from institutional factors and concurrent changes in trauma care. Importantly, future research should separately assess individual operator experience, institutional case volume, and trauma-system coordination, as these may represent distinct components of the learning process. Prospective evaluation of structured simulation, multidisciplinary training, and standardized implementation protocols may help determine whether these strategies improve performance or reduce the clinical exposure required to achieve consistent proficiency.

### 4.5. Limitations

This review has several limitations. First, all included studies were observational, and none used a controlled design specifically intended to evaluate the effect of accumulated experience. Therefore, the observed associations between experience and improved outcomes cannot be interpreted as causal and may have been influenced by concurrent changes in patient selection, trauma-system organization, device technology, and resuscitative practice. Second, REBOA was performed relatively infrequently in most settings, and several studies included small samples or limited numbers of procedures per operator or institution. This reduced the ability to estimate stable learning-curve thresholds and may have increased the influence of individual cases on the results. Furthermore, substantial heterogeneity in patient populations, outcome definitions, and analytical approaches, together with overlapping populations and study periods in some registry-based studies, precluded calculation of a meaningful average or pooled mortality estimate. Third, the inclusion of calendar-time studies broadened the evidence base but also increased conceptual heterogeneity. Because calendar year is an indirect and potentially misclassified measure of accumulated experience, these studies may overestimate or underestimate the contribution of learning to temporal outcome improvement. Four [38,39,41,42] of the seven included studies provided only indirect temporal evidence, whereas only three [11,40,43] directly modeled cumulative case experience. Accordingly, the review’s ability to characterize a true procedural learning curve was limited. Fourth, procedural efficiency and procedural safety, which were the prespecified primary outcomes, were inconsistently defined and reported across the included studies. This limited direct comparison and precluded quantitative synthesis. Fifth, the reported proficiency thresholds were derived from different outcomes, analytical methods, and levels of assessment. A threshold based on technical difficulty, procedural time, or mortality may represent different aspects of proficiency and should not be considered interchangeable. Sixth, several reports analyzed partially overlapping registry or institutional cohorts; therefore, the seven reports should not be interpreted as seven independent patient populations. Seventh, the review was restricted to English-language publications, and relevant studies published in other languages may have been missed. Finally, a formal GRADE assessment was not performed because the included studies had heterogeneous designs, outcomes, and analytical methods and lacked a consistent comparison framework. Nevertheless, confidence in the available evidence is very limited for both direct case-based learning-curve evidence and indirect calendar-time evidence. Direct evidence was limited by the small number of studies, limited risk adjustment, heterogeneous endpoints, inconsistent findings, and lack of external validation, whereas temporal-trend studies were additionally limited by the use of calendar time as an indirect measure of accumulated experience and susceptibility to temporal confounding.

## 5. Conclusions

Direct clinical evidence regarding the REBOA learning curve remains limited to three case-based studies that identified heterogeneous, outcome-specific inflection points using different analytical methods and levels of assessment. These findings do not establish a reproducible or generalizable case-volume threshold for proficiency. Four additional registry-based studies reported improved outcomes over calendar time, but these findings represent indirect evidence that may reflect broader changes in patient selection, devices, resuscitative practice, and trauma-system organization. This review provides an integrated clinical perspective on how REBOA proficiency may develop with accumulated experience in real-world practice. Prospective studies using direct cumulative-experience measures, standardized procedural outcomes, and risk-adjusted multilevel learning-curve methods are needed.

## Figures and Tables

**Table 1:** Characteristics and classification of included clinical studies on REBOA experience.

Study	Setting/Data Source	Design/Period	Sample/Population	Experience Level and Measure	Evidence Classification	Analysis/Risk Adjustment	Outcome(s)	Main Finding/Reported Threshold
Choi et al., 2023 [40]	Republic of Korea; single regional trauma center; institutional trauma registries	Retrospective case series; Jul 2017–Jun 2021	*n* = 31 consecutive trauma patients with successful balloon inflation; ISS 34 (25–44)	Institution; sequential case number	Direct case-based learning-curve evidence	Non-risk-adjusted CUSUM of mortality and occlusion-time failure; pre-trauma-center vs. trauma-center periods	In-hospital mortality; aortic occlusion time within 60, 90, and 120 min	Downward CUSUM inflection at case 17, indicating improved hospital-level performance.
Moore et al., 2026 [11]	USA and Canada; 8 Level I trauma centers; structured post-procedural debriefings	Prospective multicenter observational quality-improvement study; Mar 2021–Jun 2024	377 procedures overall; surgeon analysis: 251 cases by 24 surgeons; institutional analysis: 330 cases at 8 centers	Individual surgeon and institution; cumulative case number	Direct case-based learning-curve evidence	Cubic regression of technical challenges; no patient-level risk adjustment	Technical challenges in femoral access, catheter removal, and sheath management	Individual surgeon level Inflection Point: 5 cases Cubic Fit: R^2^ = 0.900, *p* < 0.001 Comparison: 0–5 cases group had a significantly higher challenge rate than subsequent groups (post hoc: *p* = 0.023) Institutional Level Inflection Point: 10 cases Cubic Fit: R^2^ = 0.436, *p* < 0.001 Comparison: 0–10 cases group had a significantly higher challenge rate than 51–60 (*p* = 0.002) and 61–70 (*p* = 0.013) groups
Kim et al., 2026 [43]	Republic of Korea; 5 regional trauma centers; institutional trauma registries	Retrospective multicenter cohort; Dec 2015–Dec 2021	*n* = 251 consecutive REBOA cases; ISS 34 (25–43)	Institution; sequential case number from each center’s first case	Direct case-based learning-curve evidence	RA-CUSUM using LASSO-predicted exsanguination mortality with V-mask; additional procedural-time CUSUMs	Mortality due to exsanguination; placement, occlusion, door-to-balloon, and injury-to-admission times	Risk-adjusted mortality improvement after 5–34 cases in 3 of 5 centers; no consistent improvement in 2 centers; time CUSUMs showed no significant change. RA-CUSUM fluctuations exceeded V-mask control limits
Aoki et al., 2020 [38]	Japan; nationwide Japan Trauma Data Bank	Retrospective nationwide cohort; 2004–2015	*n* = 633 REBOA patients; ISS 33–34 across calendar periods	National; successive calendar periods (2004–2007, 2008–2011, 2012–2015)	Indirect experience-related temporal evidence	Mixed-effects logistic regression adjusted for patient characteristics, trauma severity, and therapeutic choices	In-hospital survival; survival during the first 2 days	In-hospital survival increased from 39% to 49% and 60% (*p* = 0.001); late vs. early aOR 2.976 (95% CI = 1.615–5.482). No case-volume threshold.
Bukur et al., 2021 [39]	USA; multicenter AAST AORTA registry	Retrospective analysis of a prospective observational registry; Jan 2014–Nov 2018	*n* = 568 REBOA cases (1458 aortic occlusions overall); ISS 34 (24–43)	Registry/system; calendar year	Indirect experience-related temporal evidence	Multivariable logistic regression with procedure year and mortality risk factors	In-hospital mortality; utilization and technique changes; complications	Adjusted REBOA mortality decreased per year (aOR 0.78, 95% CI 0.62–0.97, *p* = 0.024) accompanied by changes in devices and deployment practice. No threshold.
Hoshi et al., 2024 [41]	Japan; nationwide Japan Trauma Data Bank	Retrospective nationwide descriptive cohort; 2004–2021	*n* = 2557 REBOA patients; ISS 34 (25–45)	National/facility; calendar year	Indirect experience-related temporal evidence	Annual trend analyses and logistic regression adjusted for TRISS probability of survival	In-hospital mortality; REBOA case and facility counts; injury severity	Mortality decreased from 91.3% to 50.9%, with declining adjusted odds over time. No case-volume threshold.
Rayzah et al., 2026 [42]	USA; National Trauma Data Bank	Retrospective cohort; 2019–2022	*n* = 404 abdominal trauma patients undergoing REBOA; ISS 17 (10–25)	National; calendar year	Indirect experience-related temporal evidence	Descriptive annual trend; multivariable mortality-predictor model, but the temporal trend was not risk-adjusted	In-hospital mortality; zone-specific outcomes; transfusion and complications	Mortality decreased from 58% to 36%; the temporal trend could not isolate accumulated experience. No threshold.

Note 1. Direct evidence used sequential case number or cumulative procedural volume; indirect evidence used calendar time as a proxy and was not used to derive proficiency thresholds. Note 2. Reported thresholds are study-specific outcome inflection points, not validated competency standards. Values are median (interquartile range) where applicable. Abbreviations: AAST, American Association for the Surgery of Trauma; AO, aortic occlusion; aOR, adjusted odds ratio; AORTA, Aortic Occlusion in Resuscitation for Trauma and Acute Care Surgery; CI, confidence interval; CUSUM, cumulative sum; ISS, Injury Severity Score; LASSO, least absolute shrinkage and selection operator; RA-CUSUM, risk-adjusted cumulative sum for mortality; REBOA, resuscitative endovascular balloon occlusion of the aorta; TRISS, Trauma and Injury Severity Score.

**Table 2:** Methodological quality of the included studies assessed with the Newcastle–Ottawa Scale (NOS) for cohort studies.

Study (Author, Year)	S1 Representativeness of the Exposed Cohort	S2 Selection of the Non-Exposed Cohort	S3 Ascertainment of Exposure	S4 Outcome Not Present at Start	C1 Control for the Most Important Factor	C2 Control for Any Additional Factor	O1 Assessment of Outcome	O2 Follow-Up Long Enough for Outcome	O3 Adequacy of Follow-Up
Aoki et al., 2020 [38]	1	N/A	1	1	1	1	1	1	1
Bukur et al., 2021 [39]	1	N/A	1	1	1	1	1	1	1
Choi et al., 2023 [40]	1	N/A	1	1	0	0	1	1	1
Hoshi et al., 2024 [41]	1	N/A	1	1	1	0	1	1	1
Moore et al., 2026 [11]	0	N/A	1	1	0	0	0	1	1
Rayzah et al., 2026 [42]	1	N/A	1	1	0	0	1	1	1
Kim et al., 2026 [43]	1	N/A	1	1	1	1	1	1	1

Note: The Newcastle–Ottawa Scale (NOS) for cohort studies was applied. N/A, not applicable. S2 was considered not applicable when the study did not include an independently selected non-exposed cohort; earlier cases or periods within the same cohort were not considered a separate non-exposed cohort. Each numbered item scores 1 if the criterion is met and 0 if it is not; Comparability comprises two items (C1, C2) and may therefore score up to 2. One point is equivalent to one star in the original instrument.

## Data Availability

All data analyzed during this study are included in this published article and its Supplementary Information files.

## Supplementary Materials

The following supporting information can be downloaded at: https://www.mdpi.com/article/10.3390/jcm15186959/s1, Table S1: Searching strategy; Table S2: Studies excluded after full-text assessment; PRISMA checklist [44], AMSTAR2 checklist.

## Abbreviations

The following abbreviations are used in this manuscript:

CUSUM	Cumulative sum
MEDLINE	Medical Literature Analysis and Retrieval System Online
MeSH	Medical Subject Headings
NCTH	Non-compressible torso hemorrhage
NOS	Newcastle–Ottawa Scale
PRISMA	Preferred Reporting Items for Systematic Reviews and Meta-Analyses
PROSPERO	International Prospective Register of Systematic Reviews
RA-CUSUM	Risk-adjusted cumulative sum
REBOA	Resuscitative endovascular balloon occlusion of the aorta
RoB 2	Revised Cochrane risk-of-bias tool for randomized trials
ROBINS-I	Risk of Bias in Non-randomized Studies of Interventions
UK-REBOA	UK Resuscitative Endovascular Balloon Occlusion of the Aorta trial
