# Supplementary material for: Clinical Learning-Curve Evidence and Experience-Related Temporal Trends of Resuscitative Endovascular Balloon Occlusion of the Aorta in Patients with Trauma: A Systematic Review
Source: J Clin Med. 2026 Sep 8;15(18):6959. doi: 10.3390/jcm15186959 (PMC13607243; doi:10.3390/jcm15186959)
Supplement: Supplementary file 1 [file jcm-15-06959-s001.zip › jcm-4522138-supplementary final.pdf]

Supplementary Table S1. Searching strategy

Database	Query	Result	Date
PubMed	#1 trauma*[tiab] OR "Wounds and Injuries"[MeSH Terms] OR "Hemorrhagic Shock"[tiab] OR Hemorrhag*[tiab] OR Shock[tiab] OR Exsanguination[tiab]	1,844,029	2026-04-24
	#2 "Resuscitative Endovascular Balloon Occlusion of the Aorta"[tiab] OR REBOA[tiab] OR "Aortic balloon occlusion"[tiab] OR "Endovascular balloon occlusion"[tiab]	1,339	2026-04-24
	#3 #1 AND #2	1,011	2026-04-24
EMBASE	#1 'trauma'/exp OR 'trauma' OR 'injury'/exp OR 'injury' OR trauma*:ti,ab,kw OR 'hemorrhagic shock':ti,ab,kw OR hemorrhag*:ti,ab,kw OR shock:ti,ab,kw OR exsanguination:ti,ab,kw	4,606,916	2026-04-24
	#2 'resuscitative endovascular balloon occlusion of the aorta':ti,ab,kw OR 'reboa':ti,ab,kw OR 'aortic balloon occlusion':ti,ab,kw OR 'endovascular balloon occlusion':ti,ab,kw	1,715	2026-04-24
	#3 #1 AND #2	1,461	2026-04-24
Cochrane	#1 trauma*:ti,ab,kw OR [mh "Wounds and Injuries"] OR "Hemorrhagic Shock":ti,ab,kw OR Hemorrhag*:ti,ab,kw OR shock:ti,ab,kw OR Exsanguination:ti,ab,kw	125,613	2026-04-24
	#2 "Resuscitative Endovascular Balloon Occlusion of the Aorta":ti,ab,kw OR "REBOA":ti,ab,kw OR "Aortic balloon occlusion":ti,ab,kw OR "Endovascular balloon occlusion":ti,ab,kw	51	2026-04-24
	#3 #1 AND #2	39	2026-04-24

Supplementary Table S2. Studies excluded after full-text assessment

No.	Reason for exclusion	Study (Author, Year)	Country	Study design	Participants / Population	Model / Setting
1	Non-clinical study (simulation, cadaver, or animal model)	Brenner et al., 2014	USA	Prospective single-center educational (simulation) study	13 trauma and critical care faculty members (endovascular-naive)	Vascular Intervention System Training virtual-reality simulator (BEST course)
2	Non-clinical study (simulation, cadaver, or animal model)	Villamaria et al., 2014	USA	Curriculum development and validation study (pilot courses)	16 trauma surgeons (4 pilot courses × 4 participants)	Virtual-reality endovascular simulator and live-animal (porcine) laboratory
3	Non-clinical study (simulation, cadaver, or animal model)	Teeter et al., 2017	USA	Prospective observational skill-acquisition study	4 US Army Special Operations Command personnel (2 non-surgeon physicians, 1 physician assistant, 1 combat medic)	Simulation (BEST course task trainer)
4	Non-clinical study (simulation, cadaver, or animal model)	Pasley et al., 2018	USA	Prospective simulation study	11 US Air Force independent duty medical technicians without prior endovascular experience	Simulation (task trainer)
5	Non-clinical study (simulation, cadaver, or animal model)	Ross & Redman, 2018	USA	Skill-acquisition and feasibility study	2 board-certified military emergency medicine physicians without prior endovascular exposure	Perfused human cadaver model in field, ambulance, and UH-60 aircraft settings

6	Non-clinical study (simulation, cadaver, or animal model)	Borger van der Burg et al., 2018 (J R Army Med Corps)	The Netherlands / Sweden (EVTM workshop, Örebro)	Prospective feasibility / training study	21 workshop participants with no or limited endovascular experience	Live-tissue (porcine) – simulator hybrid model
7	Non-clinical study (simulation, cadaver, or animal model)	Borger van der Burg et al., 2018 (JSOM)	The Netherlands (EVTM workshop, Leiderdorp)	Prospective feasibility / skill-acquisition training study	10 Special Operations Forces medics, 5 combat nurses, 4 military nonsurgeon physicians, and 4 military surgeons (nonvascular; control group)	Simulation – REBOA Access Task Trainer (RATT; Prytime Medical) at an EVTM workshop
8	Non-clinical study (simulation, cadaver, or animal model)	Brede et al., 2019	Norway	Descriptive evaluation of an educational programme	7 physicians and 3 paramedics of a national air ambulance base	Simulation mannequin, simulated prehospital setting
9	Non-clinical study (simulation, cadaver, or animal model)	Burkard et al., 2020	USA	Quality-improvement project (tool development + simulation course)	Providers and support staff involved in REBOA deployment	Standardized simulation course; case evaluation (review) tool
10	Non-clinical study (simulation, cadaver, or animal model)	Hatchimonji et al., 2020	USA	Prospective educational cohort (skill-retention) study	13 participants (10 trauma faculty, 3 fellows)	Formal REBOA course with didactics and hands-on simulation
11	Non-clinical study (simulation, cadaver, or animal model)	Onufer et al., 2020	USA	Curriculum development and evaluation study	60 surgical residents (41 and 36 completed surveys across 2 academic years)	Multimodal curriculum (lectures, simulation labs); a REBOA training session is one component
12	Non-clinical study (simulation, cadaver, or animal model)	Park et al., 2022	USA (Level I trauma center)	Prospective observational simulation-based training study	25 surgical trainees (PGY-2 and PGY-3)	Monthly hands-on training and class-based evaluation on a perfused trainer
13	Non-clinical study (simulation, cadaver, or animal model)	Engberg et al., 2023	Denmark / Sweden / Norway	Prospective multicenter simulation-based trial (validity evidence)	Physicians of differing clinical experience levels	Simulation model with an assessment tool
14	Non-clinical study (simulation, cadaver, or animal model)	Studer et al., 2024	USA	Prospective pilot educational study	11 emergency medicine residents	12-hour course with task trainers and perfused cadavers; 4-month retention assessment
15	Not original article	Engberg et al., 2020	Denmark	Systematic review	16 included studies (189 trainees in full-text studies)	Review of training and assessment studies (mainly simulation-based)
16	No learning-curve or cumulative-	Romagnoli et al., 2017	USA (single institution)	Retrospective single-center review of	Trauma patients undergoing REBOA or resuscitative	Clinical (trauma bay), video-review time analysis

	experience analysis			videographic recordings	thoracotomy with aortic cross-clamping	
17	No learning-curve or cumulative-experience analysis	Theodorou et al., 2020 (JEVTM)	USA (Level I trauma center)	Retrospective single-center implementation review	Trauma patients during the first year after REBOA introduction	Clinical (program implementation, opportunities-for-improvement review)
18	No learning-curve or cumulative-experience analysis	Theodorou et al., 2020 (J Surg Res)	USA (multicenter, AORTA registry)	Retrospective multicenter registry study	Adults (≥18 yr) undergoing REBOA, 11/2013–01/2018	Clinical (registry); hospitals grouped as high-, mid-, and low-volume
19	No learning-curve or cumulative-experience analysis	Matsumoto et al., 2020	Japan (Japan Trauma Data Bank, 2004–2014)	Retrospective nationwide registry study	Adult patients with pelvic fracture in shock	Clinical (registry); comparison of AG/AE, external fixation, and REBOA
20	No learning-curve or cumulative-experience analysis	Gorman et al., 2021	USA (multicenter, AAST AORTA registry)	Retrospective multicenter registry study (volume-stratified)	Adults undergoing REBOA; institutions stratified by REBOA deployment volume	Clinical (registry); centers grouped as low- (<10), average- (11–30), and high-volume (>30)
21	No learning-curve or cumulative-experience analysis	Chang et al., 2023	Republic of Korea (single regional trauma center)	Before–after (pre-course vs. post-course) cohort study	Trauma patients undergoing REBOA, Aug 2016 – Feb 2018	Clinical, with an educational (ET-REBOA) course as the exposure
22	No learning-curve or cumulative-experience analysis	Jansen et al., 2023	United Kingdom (16 major trauma centers)	Pragmatic bayesian randomized clinical trial	Patients ≥16 yr with exsanguinating hemorrhage, Oct 2017 – Mar 2022	Clinical (randomized comparison of REBOA vs. standard care alone)
23	No learning-curve or cumulative-experience analysis	Roderick et al., 2023	USA (Level I trauma center)	Retrospective single-center descriptive implementation study	Trauma patients with non-compressible torso hemorrhage over 7 months	Clinical (program implementation with multidisciplinary guidelines)
24	No learning-curve or cumulative-experience analysis	Creel & Mantooth, 2024	USA (rural Level II trauma center)	Retrospective comparison with a matched historical control group	Trauma patients before vs. after REBOA implementation	Clinical, with an in-house low-cost training program

Note: Twenty-four reports were excluded after full-text assessment. Studies are grouped by primary reason for exclusion and, within each group, listed in chronological order of publication. Each report was assigned a single primary reason, although several met more than one exclusion criterion. Engberg et al. (2020) is a systematic review and its reference list was hand-searched for additional eligible reports.

Abbreviations: AAST, American Association for the Surgery of Trauma; AG/AE, angiography/angioembolization; AORTA, Aortic Occlusion in Resuscitation for Trauma and Acute Care Surgery; BEST, Basic Endovascular Skills for Trauma; ET-REBOA, Endovascular Training for REBOA; EVTM, endovascular resuscitation and trauma management; JEVTM, Journal of Endovascular Resuscitation and Trauma Management; JSOM, Journal of Special Operations Medicine; PGY, postgraduate year; REBOA, resuscitative endovascular balloon occlusion of the aorta; UH-60, Black Hawk helicopter
